# Copper Oxide Precipitates in NBS Standard Reference Material 482

**DOI:** 10.6028/jres.107.053

**Published:** 2002-12-01

**Authors:** Eric S. Windsor, Robert A. Carlton, Greg Gillen, Scott A. Wight, David S. Bright

**Affiliations:** National Institute of Standards and Technology, Gaithersburg, MD 20899-8371; Elan Drug Delivery, Inc., King of Prussia, PA 19406; National Institute of Standards and Technology, Gaithersburg, MD 20899-8371

**Keywords:** copper-gold alloy, electron probe microanalysis, metallography, NBS Standard Reference Material 482, oxide inclusions, sample preparation, secondary ion mass spectrometry

## Abstract

Copper oxide has been detected in the copper containing alloys of NBS Standard Reference Material (SRM) 482. This occurrence is significant because it represents heterogeneity within a standard reference material that was certified to be homogeneous on a micrometer scale. Oxide occurs as elliptically to spherically shaped precipitates whose size differs with alloy composition. The largest precipitates occur in the Au20-Cu80 alloy and range in size from submicrometer up to 2 μm in diameter. Precipitates are observed using light microscopy, electron microscopy, and secondary ion mass spectrometry (SIMS). SIMS has demonstrated that the precipitates are present within all the SRM 482 wires that contain copper. Only the pure gold wire is precipitate free. Initial results from the analysis of the Au20-Cu80 alloy indicate that the percentage of precipitates is less than 1 % by area. Electron probe microanalysis (EPMA) of large (2 μm) precipitates in this same alloy indicates that precipitates are detectable by EPMA and that their composition differs significantly from the certified alloy composition. The small size and low percentage of these oxide precipitates minimizes the impact that they have upon the intended use of this standard for electron probe microanalysis. Heterogeneity caused by these oxide precipitates may however preclude the use of this standard for automated EPMA analyses and other microanalysis techniques.

## 1. Introduction

**Fig. 1:**
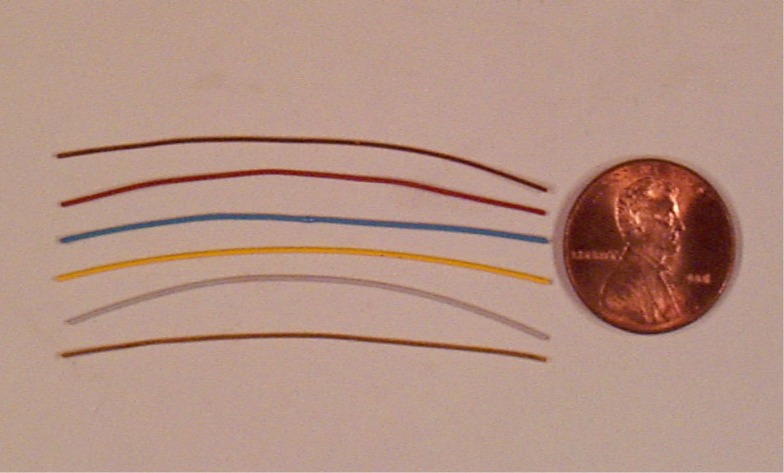
SRM 482. Copper-Gold binary alloys for microanalysis. The SRM is a set of six wires. Wires are approximately 5 cm long and 0.5 mm in diameter. Each wire has a different composition within the copper-gold binary system.

Standard Reference Material (SRM) 482 was issued in 1969 by the National Bureau of Standards (NBS)^1^. It has been continuously available to the public for 33 years. The standard consists of a set of six wires (Fig. 1). Each wire is of a different composition within the copper-gold binary alloy system. Two uncoated wires represent the pure end member compositions of pure copper (Cu) and pure gold (Au). The remaining four wires are alloys with nominal compositions varying in steps of 0.2 mass fraction. For identification purposes, each alloy wire was coated with a different colored paint. Their composition and color is as follows: Au20-Cu80 (red), Au40-Cu60 (blue), Au60-Cu40 (yellow), and Au80-Cu20 (gray). Each wire is approximately 5 cm long and 0.5 mm in diameter. SRM 482 was issued specifically as a standard for microanalysis. Therefore, each wire was certified for both chemical composition and homogeneity on a micrometer scale (Appendix A).

At the time that SRM 482 was issued, Heinrich [1] reported that there was concern about the usefulness of the electron probe microanalyzer (EPMA) as a quantitative tool for chemical analysis. Often, analyses performed on the same material by different experienced investigators varied in excess of 10 % relative. Factors contributing to these high relative errors included systematic errors in addition to errors resulting from the application of different matrix correction procedures. Additionally, Heinrich determined that a major contributing factor to these errors was the lack of standard materials of accurately known chemical composition and microscopic homogeneity [2]. SRM 482 was then issued in response to this need for chemically characterized homogeneous standards.

Since the time it was issued, SRM 482 has been well accepted and widely used throughout the microanalysis community. Uses include the evaluation and modification of matrix correction procedures [3–7], evaluation of EPMA instrument performance [8], and investigation of systematic errors associated with electron probe microanalysis [9]. To this day, SRM 482 remains one of a very limited number of standard reference materials available from NIST that is certified to be homogeneous on a microscopic scale [10].

Recently, homogeneity of SRM 482 has been questioned. Carlton reported the occurrence of spots on metallographically prepared surfaces of the Au20-Cu80 and the Au60-Cu40 wires [11]. The presence of these spots was then verified by independent preparations performed at NIST [12].

The occurrence of these spots raises the following questions:

1. Do the spots represent heterogeneity within the wires or are they artifacts that were created during metallographic sample preparation?
2. What is the chemical composition of these spots?
3. Does the occurrence of these spots affect the use of SRM 482 as a standard for electron probe microanalysis?
4. If these spots represent heterogeneity within the wires, then why were they not detected prior to certification and why have they not been reported in 33 years since SRM 482 was issued?

The purpose of this manuscript is to report results from work that is in progress to answer these questions and investigate the possibility of heterogeneity within SRM 482. Our investigation centers on the Au20-Cu80 wire because the spots are largest and appear most abundant in this composition. Therefore, if the spots do significantly affect the microanalysis of SRM 482, we would expect this effect to be most noticeable in the Au20-Cu80 wire.

## 2. Samples, Preparation, and Initial Observations

When the wires of SRM 482 were manufactured, they were drawn as one continuous wire [1]. Wire length was approximately 150 m. Extensive homogeneity testing was performed prior to the issuance of SRM 482 as a standard for microanalysis [1]. This original homogeneity testing is summarized in Appendix B and Fig. 12. Historically, there has been only one issue of SRM 482. Therefore, wire sets (SRM 482) obtained today should be equivalent to those purchased 30 years ago.

### 2.1 Samples

At NIST, three different sets of SRM 482 were used to investigate the reported occurrence of spots on metallographically prepared surfaces of these wires. Two sets were new boxes of SRM 482 obtained from the office of The Standard Reference Materials Program (SRMP). The third set was donated by Carlton and consisted of pieces of wire from the set that was originally reported to contain these spots [11]. The wire sets obtained from SRMP represent random samples of SRM 482 that are currently available for sale to the general public. For the remainder of this manuscript, wire sets purchased from SRMP will be referred to as set A and set B. Carlton’s wires will be referred to as set C.

### 2.2 Sample Preparation

**Fig. 2:**
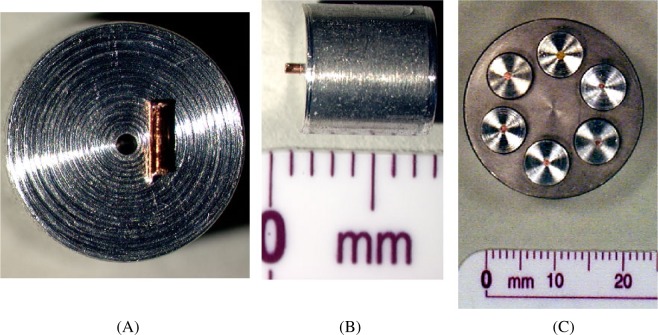
SRM 482 wires are mounted into aluminum bullets prior to grinding and polishing. (A) Using a #76 drill bit, a hole slightly larger than the diameter of the wire is drilled into each bullet. Holes are drilled 1.5 mm deep. Wire pieces are cut to a length of 2 mm. (B) Wire pieces are inserted into drilled holes. The wire is then impressed down into the holes using a mounting press. (C) Six bullets, (one for each wire composition in SRM 482) with wires impressed, are mounted into a 25.4 mm (1 in) diameter bullet holder for grinding and polishing.

At NIST, initial preparations consisted of one metallographically polished mount from each set (A, B, and C). The wires were mounted in aluminum so that the samples would be completely conductive in the electron beam without the need to add a conductive surface coating such as carbon. Each wire was mounted into a 6.3 mm (1/4 in) diameter aluminum rod that had been previously cut into pieces referred to as “bullets.” Bullets are approximately 6 mm to 7 mm long. In order to mount the wires into the bullets, a single hole was drilled into one end of each bullet (Fig. 2). A #76 drill bit produced a hole just slightly larger than the diameter of the wire itself. Holes were drilled to a depth of 1.5 mm and wires were cut into pieces approximately 2 mm long. The wire pieces were then inserted into the holes and press fit down into the aluminum bullets using a mounting press. All six wires from each set were prepared in this manner. These six bullets were then inserted into a single bullet holder 25.4 mm (1 in) in diameter (Fig. 2). At this point the wires were ready for grinding and polishing.

Samples were ground and polished using a Buehler Ecomet 3 Variable Speed Grinder/Polisher interfaced with an Automet 2 Power Head^2^. As a guide, we followed the grinding and polishing procedure recommended for the preparation of copper and copper alloys by ASM International [13]. Our initial preparation procedure is listed as preparation procedure #1 in Appendix C.

### 2.3 Light Microscope Observations

**Fig. 3:**
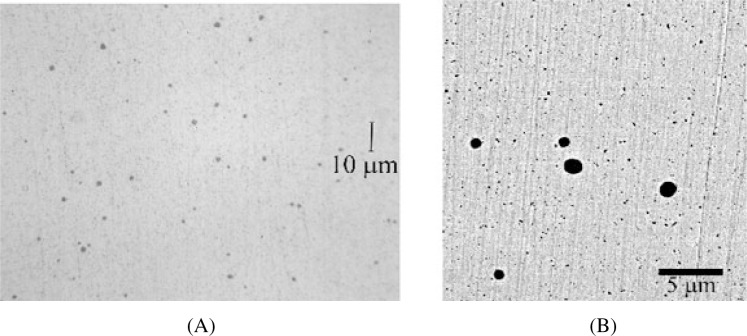
Spots observed in Au20-Cu80 wire. (A) Light microscope image. (B) Backscatter electron image. Note the occurrence of the impressed diamond abrasives (≤ 0.5 μm).

When we analyzed our preparations using the light microscope, our observations were similar to those reported by Carlton [11]. Spots were observed in the Au20-Cu80 wire (Fig. 3) from all three sets (A, B, and C) of SRM 482. Spots were also observed in pure copper, Au40-Cu60 and Au60-Cu40 wires in at least one, but not all three of the prepared sets. No spots were observed by light microscopy in the Au80-Cu20 or the pure gold wires.

The observed spots are circular in cross-section and dull gray in color when observed in bright field reflected light (Fig. 3A). In dark field and crossed polarized light, spots are often, but not always, bright red in color. In some preparations we observed that the spots could not be seen in polarized or darkfield illumination even though they were easily observed in bright field illumination. Spot size was observed to differ with wire composition. Spots are largest and most easily observed in the Au20-Cu80 wire, where they range in size from sub-micrometer up to approximately 2 μm in diameter. In wires of other composition, the spots are approximately 1 μm or less in diameter. Spots of this size are difficult to distinguish from diamond abrasives that have become impressed into these wires during sample preparation (Fig. 3B).

After observing spots in our preparations, we obtained and examined one of the original NBS standard mounts. The Microanalysis Research Group at NBS/NIST has used this standard mount for the past 3 decades. It was metallographically prepared and repolished as needed over the years by workers other than the current authors. Interestingly, no spots were observed when this original preparation was examined by both light and scanning electron microscopy.

Did the spots that we observed represent heterogeneity or were they artifacts from the sample preparation procedure? It was evident that additional work was required to answer this question.

## 3. Auger Analysis

Carlton reported that the characteristics of the observed spots were consistent with the properties of copper oxide [11]. His conclusion was based upon the facts that the spots are red in color when viewed by cross-polarized light microscopy and that EPMA analysis showed that they contain copper and oxygen. The red coloration observed with cross-polarized light microscopy is consistent with the properties of cuprous oxide Cu_2_O. At NIST however, we observed that these spots are not always red in color when viewed with cross-polarized illumination. Also, some uncertainty remained as to whether oxygen detected by electron probe microanalysis originated within the spots themselves or whether it was due to a thin oxide coating covering the spots.

**Fig. 4:**
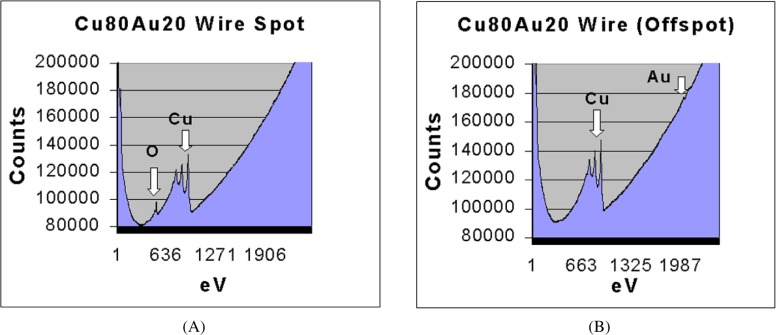
Auger Spectra from the Au20-Cu80 wire. (A) Analysis taken on one of the observed spots. The spot analysis contains copper and oxygen only. (B) Analysis from the wire itself contains copper and gold only.

We used Auger analysis to verify that the composition of these spots was copper oxide and also to demonstrate that oxygen detected by EPMA analysis originated from the spots and was not due to an oxide surface coating. Auger analyses were performed using a JEOL 7830F Scanning Auger Microprobe with a thermally assisted field emitter electron source and an ultra high vacuum specimen chamber. This Auger microprobe is equipped with an argon ion gun that is used to sputter clean the surface of the sample prior to analysis. The Au20-Cu80 wire was analyzed after the surface was thoroughly sputtered to remove any oxide film. Several spots were analyzed. These spots contained copper and oxygen (Fig. 4). Analysis of the wire itself (off spot in Fig. 4) detected only copper and gold. These analyses verified that the composition of the observed spots was copper oxide.

## 4. Secondary Ion Mass Spectrometry (SIMS)

After the spots were identified as copper oxide, it became important to investigate the distribution of these oxides with depth in our polished samples. If the copper oxide spots were preparation artifacts, then we would expect them to be concentrated at the surface and decrease in number with depth (distance from the surface). We chose secondary ion mass spectrometry (SIMS) as a tool to investigate this distribution of copper oxide spots with depth. In SIMS, energetic ions bombard the sample and sputter down through the surface preparation into regions of the sample unaltered by sample preparation.

**Fig. 5:**
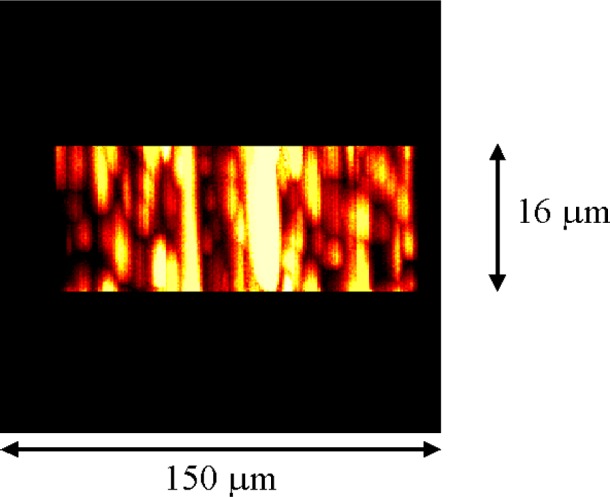
Projected three-dimensional SIMS oxygen ion image of the Au20-Cu80 wire. This image was formed by collecting and stacking approximately 90 images taken at regular intervals while sputtering down through the polished surface of the wire. The stacked images were then projected using the “nearest point” projection method (Scion Image available at www.scioncorp.com) so as to be viewed from the side. This produced a three-dimensional image of the sputtering event. Sputtering depth was approximately 16 μm. Spherical oxides appear elongated in this image due to the combination of an elongated depth scale and different sputtering rates between the oxides and the metallic wire.

**Fig. 6:**
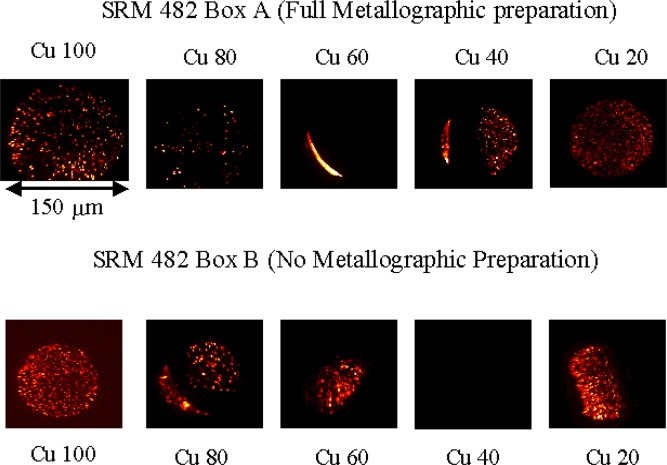
SIMS oxygen ion images for two different samples of SRM 482. The upper row of images was prepared from box A. These samples received a full metallographic preparation according to preparation procedure #1 in Appendix B. The lower row of images was prepared from box B. These wires were cut (sheared) using a razor blade and received no metallographic sample preparation. Note that inclusions are observed in the Au40-Cu60 wire from box B but not from box A. Similarly, inclusions are observed in the Au60-Cu40 wire from box A but not from box B.

SIMS analyses were performed using a Cameca IMS 4F ion microscope. Cesium ions were used to bombard the samples at an impact energy of 14.5 keV. Negative secondary ions were detected and the typical field of view was 150 μm. Images were acquired with a 14 bit scientific grade slow scan CCD camera using integration times of 10 s to 15 s. The first sample analyzed was a metallographic preparation of the Au20-Cu80 wire from set A. During analysis, a crater was sputtered down through the polished cross-sectional surface of the wire. Crater depth was approximately 16 μm. While sputtering, a series of oxygen ion images were collected as a function of increasing depth below the sample surface. In Fig. 5, these images are stacked and projected to obtain a three dimensional view that represents our observations during the sputtering process. In this figure, spherical copper oxides appear as elongated vertical streaks due to the combination of an elongated vertical (depth) scale and different sputtering rates between the oxide and the metal wire. Small (submicrometer) oxide spheres were sputtered away rapidly while larger spheres (approximately 2 μm in diameter) remained present during the entire sputtering event (represented by vertical streaks from top to bottom in Fig. 5). Oxides were continually encountered and exposed at increasing depth within this sample. The concentration of oxide precipitates remained approximately constant throughout the sputtering event. In the same experiment, the remaining wires from set A were analyzed. All wires except the pure gold and the Au40-Cu60 contained copper oxide precipitates (Fig. 6). For each wire that contained oxides, we verified that these oxides were distributed with depth in our metallographically prepared cross-sections. The results of the SIMS experiment indicate that copper oxides are precipitates within the wires themselves and not sample preparation artifacts. To verify this conclusion, wires from set B were also analyzed. These wires however, were not metallographically prepared. Instead, cross-sections were prepared by cutting (shearing) the wires with a razor blade. SIMS analysis of the sheared surfaces showed that all of the wires except the Au60-Cu40 and the pure gold contained oxide precipitates. In all cases, these precipitates were once again observed to be distributed with depth in these wires (Fig. 6).

It is important to note that the pure copper wire contains oxide precipitates (Fig. 6). This indicates that a possible source of precipitates in SRM 482 may have been the pure copper starting material that was used to manufacture these alloys. Another observation of importance is that copper oxide precipitates may not be evenly distributed along the lengths of the wires. This can be seen in Fig. 6 where oxide precipitates are present in the Au40-Cu60 wire in set B but not in this same wire composition in set A. Similarly, precipitates are observed in the Au60-Cu40 wire in set A but not in set B.

In summary, SIMS provided useful qualitative information for analyzing the wires in SRM 482. The technique is extremely sensitive for detecting oxygen (typical detection limits in the parts per million range). Impressed diamond abrasives did not interfere with SIMS analyses as they do with light and electron microscopy. This is important because it allowed us to detect oxide precipitates in alloys such as Au80-Cu20 that were previously thought to be oxide free.

## 5. Image Analysis and EPMA

Once we determined that the spots in SRM 482 were oxide precipitates rather than sample preparation artifacts, it became important to determine their abundance and also to measure the effect that they have on EPMA analysis.

### 5.1 Image Analysis

**Fig. 7:**
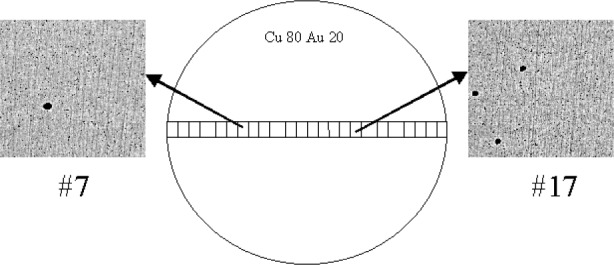
Image analysis procedure used to determine the area percentage of copper oxide inclusions in the Au20-Cu80 wire. Images were collected across a randomly selected diameter of the wire. The 7th and 17th image collected are shown. The area percentage copper oxide was determined to be between 0.2 % and 0.9 %. Scale: Wire diameter is approximately 0.5 mm. Image width for #7 and #17 is approximately 24 μm.

Image analysis was used to estimate the area percentage of copper oxide precipitates in the Au20-Cu80 wire. A series of backscatter electron images was collected across a randomly selected diameter of the wire (Fig. 7). Twenty-four images were collected, each at a magnification of 5000×. Backscatter imaging mode was selected because there is considerable contrast between copper oxide precipitates and the surrounding wire. Precipitates appear as dark spots against the lighter background of the wire (Fig. 3 and Fig. 7). After image collection, we used the Lispix^3^ image analysis software to estimate the concentration of the copper oxide precipitates [14].

During image analysis, it was difficult to differentiate between copper oxide precipitates and impressed diamond abrasives (Fig. 3 and Fig. 7). Since the average atomic number of copper oxide is considerably higher than that of diamond, we tried to differentiate between these two phases by simply adjusting instrument contrast during image acquisition. As instrument contrast is increased, copper oxide should become lighter in color (or gray) before the impressed diamond abrasive particles. For this application, the technique did not work. As EPMA contrast was increased, the thin edges of the diamond abrasive particles allowed transmittance of backscattered electrons from the wire below, and thereby rendered this technique ineffective.

Consequently, particle size was used as the criterion to differentiate between copper oxide and impressed diamond abrasives. Although particle differentiation based solely on size is not ideal, it did allow us to estimate both a minimum and maximum area percentage for the copper oxide precipitates. Since 0.5 μm diamond abrasive was used in the final preparation step, particles less than or equal to 0.5 μm were arbitrarily considered to be abrasive while particles larger than 0.5 μm were considered to be copper oxide. For the sample of Au20-Cu80 analyzed, the minimum area percentage was determined to be 0.2 % while the maximum was 0.9 %. Therefore, the true area percentage of copper oxide in this cross-section is between the values of 0.2 % and 0.9 %.

### 5.2 EPMA

The electron probe microanalyzer was used to determine whether copper oxide precipitates have a measurable effect on this standard. Data was collected using a JEOL 8600 electron probe microanalyzer operating at 15 kV and 30 nA. Beam diameter was approximately 1 μm. An operating voltage of 15 kV was selected as a compromise to minimize the electron excitation volume (while analyzing small precipitates) but still retain sufficient overvoltage needed to measure CuKα and AuLα radiation. Using wavelength dispersive spectrometry (WDS), Cu Kα and Au Lα x rays were measured using a lithium fluoride (LIF) diffracting crystal. A thallium acid phthalate (TAP) crystal was used to measure copper Lα x rays. Gold Mα was measured using a pentaerythritol crystal (PET). Oxygen content was calculated by difference. Even though the oxygen content was calculated by difference, a spectrometer was tuned to oxygen Kα radiation and used in a qualitative capacity to check for the presence of oxygen during the analyses. EPMA results are listed in Table 1.

EPMA analyses were collected in scanning mode for both the precipitates and the matrix of the Au20-Cu80 alloy. For precipitate analysis, we centered the precipitate image on the cathode ray tube (CRT) and then decreased the scan length (increased the magnification) until the precipitate image completely filled the CRT screen. We then adjusted the scan length to the minimum value (maximized the magnification) to assure that the beam intersected the sample surface within the boundaries of the precipitate. For analysis of the alloy, we calculated the electron range within the Au20-Cu80 wire using the Kanaya-Okayama electron range equation [15]. For 15 kV electrons, the range was determined to be 0.8 μm. Therefore, to avoid possible interference from precipitates exposed at the surface, the alloy was analyzed at distances greater than 2 μm from precipitate boundaries.

Eleven large (≈ 2 μm diameter) copper oxide precipitates were analyzed. These analyses were observed to differ significantly from NBS certified values for this alloy. The copper concentration of 0.851 mass fraction (CuKα) within the precipitates is approximately 5 % higher than the certified value of 0.798 mass fraction for this alloy composition. The gold composition of 0.07 mass fraction (AuLα) is approximately 13 % lower than the certified value of 0.201 mass fraction. These analyses indicate that heterogeneity does exist in the Au20-Cu80 wire at a level that can be measured by EPMA. The alloy itself was then analyzed to determine whether copper oxide precipitates have any effect upon the bulk composition of the wire. Thirty-six analyses were collected and the measurements were determined to be in agreement with NBS certified values for this wire. All measured x rays except the gold Mα were within two standard deviations of NBS certified values.

## 6. Discussion

Since copper oxide is readily detected by EPMA (at least in Au20-Cu80 wire), one has to question why it was not discovered during the original homogeneity testing and why it has not been reported in 33 years since this standard was issued. Before addressing this question however, we need to establish that copper oxide formed as precipitate inclusions at the time these wires were manufactured rather than having formed by oxidation or corrosion over time.

**Fig. 8:**
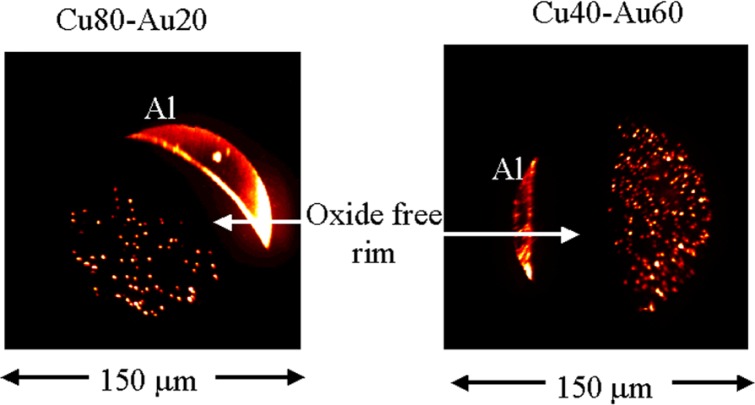
Oxygen ion SIMS images of the Au20-Cu80 and the Au60-Cu40 wires. These images show that oxides are concentrated in the interior of the wire and that there is a rim 50 μm to 100 μm wide around the exterior of the wire that is greatly reduced in oxide content.

SRM 482 has been stored under indoor air conditioned atmospheres with temperatures ranging between 20 °C and 25 °C and estimated relative humidity ranging from 20 % to 70 %. Copper exposed to indoor atmospheres oxidizes to form a tarnish that completely envelops the exposed surface [16]. This tarnish forms a protective layer [17] that retards the rate of further oxidation proportionally to the square root of time [16]. Even under conditions of outdoor exposure and extreme synthesized laboratory atmospheres (where the corrosion is extensive enough to form a removable scale), oxide corrosion still occurs as a continuous film with no evidence of any localized attack or corrosion pitting [18,19]. Therefore, it seems unlikely that the oxides in these alloys formed as a result of an oxidation reaction over time. Further evidence substantiating the conclusion that these oxides do not result from an oxidation reaction can be found in the distribution of the oxides. Oxides in the Au20-Cu80 and the Au60-Cu40 wires are observed to be concentrated in the interior of the wire (Fig. 8). The rim is nearly oxide free. Had these oxides formed by oxidation or corrosion, one would expect the oxides to be concentrated in the outer rim rather than the central core of the wire.

On the other hand, inclusions of oxide are a common occurrence in metallic copper and copper wire [20,21]. Oxygen is added to copper during the refining process to scavenge and remove impurities such as hydrogen and sulfur. Residual oxygen then occurs as cuprous oxide inclusions (Cu_2_O) [20,21]. Oxygen concentrations are normally between 2 × 10^−4^ mass fraction and 6 × 10^−4^ mass fraction (200 ppm to 600 ppm). It is important to note that even high purity copper (such as the 99.9999 % pure copper used as a starting material for these SRM wires [1]) may contain oxygen concentrations in the range of 200 ppm to 600 ppm. This is because samples are not tested for oxygen content when purity testing is performed on a metals basis.

Several reasons may explain why oxide inclusions were not detected during the original homogeneity testing of SRM 482. One possibility is that homogeneity testing was performed in oxide free regions of the wires. As we have seen from SIMS analyses, the distribution of oxides with wire length is not uniform for the Au40-Cu60 and the Au60-Cu40 wires. The same may be true for other wires in SRM 482. Even though the original homogeneity testing was extensive (appendix B), it was localized to three places along the length (150 m) of each wire [1].

**Fig. 9:**
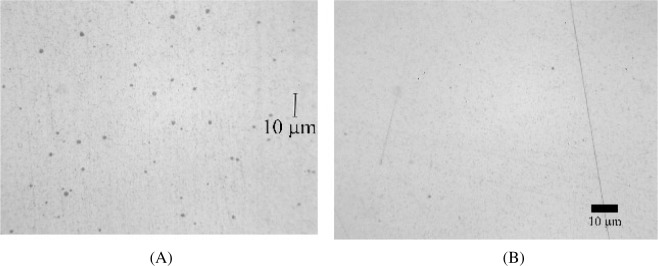
Au20-Cu80. (A) and (B) are reflected light images of the same wire that has been prepared using preparation procedure #1 and #2 respectively in Appendix C. Copper oxide inclusions are easily observed in (A) but not (B).

**Fig. 10:**
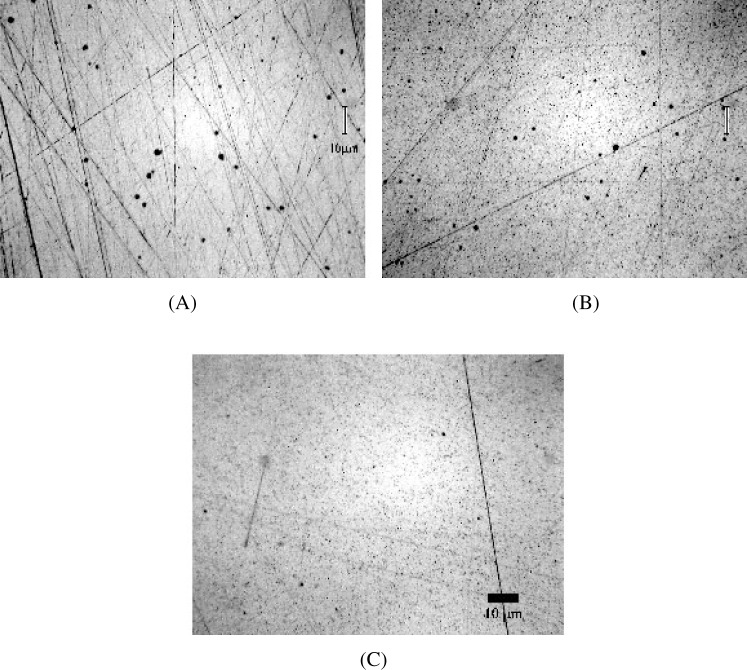
Bright field reflected light micrographs showing the surface condition after different steps in preparation procedure #2 (Appendix C). (A) Surface condition after 3 μm diamond paste on a Texmet 1000 cloth (step #3). Although scratches are abundant, oxide inclusions are easily observed and there is little evidence of impressed abrasives. (B) Surface condition after 0.1 μm diamond paste on a Texmet 1000 cloth (step #4). Impressed abrasives are now abundant. (C) Surface after 1 μm diamond paste on a Texmet 1000 cloth (step #5). Impressed abrasives are abundant. Oxide inclusions are no longer easily identified.

The most likely explanation why oxide inclusions were not observed during homogeneity testing may be due to metallographic sample preparation. During sample preparation, we observed that some metallographic procedures retained the oxide precipitates leaving them visible. Other metallographic techniques either removed the precipitates from the surface or smeared soft metal over top of them, thereby leaving them unobservable by light and electron microscopy. It is a well-known fact that nonmetallic inclusions are easily lost during preparation of metallic samples [22,23]. The physical and mechanical properties of inclusions vary considerably from those of the host metal and retention of these inclusions during metallographic preparation may require special techniques and attention to detail. As an example, Chalfant [24] was able to retain and observe lead inclusions in leaded steels only by using a carefully developed metallographic procedure in which the pH of the final alumina polishing suspension was kept exactly neutral (pH 7). Previous to these preparations, the steel industry had incorrectly assumed that lead was present as submicroscopic dispersions or as precipitated lead compounds. Samuels [22] states that, “The use of diamond abrasives is almost obligatory when examination of inclusions is important.” In addition, we have observed that the selection of polishing cloths is also important to the retention/observation of oxide inclusions in copper-gold alloys. This is illustrated in Fig. 9, where micrographs (A) and (B) are images of the same wire mount that was prepared two different ways. In micrograph (A), the wire was prepared following our preparation procedure #1 (Appendix C, Table 2). Oxide precipitates are clearly visible in this preparation. The same mount was then prepared a second time following preparation procedure #2 (Appendix C, Table 2). Oxide precipitates were not observed after this second preparation (micrograph (B) Fig. 9). Since the abrasives used in both preparation procedures are the same (silicon carbide followed by diamond paste), the only differences between the two procedures are lubricants and polishing cloths. We repeated procedure #1 using water-based lubricants and found that this change did not make a significant difference in the observation of the copper oxide precipitates. Therefore, we conclude that the choice of polishing cloths is important for the retention and observation of copper oxide inclusions when preparing SRM 482. In our preparations, oxide inclusions were lost during the final stages of sample preparation (Fig. 10). Even when we prepared the standard following procedure #2 (oxides not retained), we observed that the oxides were clearly visible after step 3 (3 μm diamond paste on Texmet 1000). Observation after step 4 (1 μm diamond paste on Texmet 1000) indicated that oxides were beginning to disappear while abrasives were becoming impressed. After step 5 (0.1 μm diamond paste on Texmet 1000), it was difficult to observe oxides in this sample.

In conclusion, care must be taken during final preparation steps in order to retain and observe oxide inclusions in SRM 482. In general, diamond abrasives on napped cloths preserve oxide inclusions while the same abrasive on unnapped chemotextile (Texmet) cloths either remove oxides from the prepared surface or smears soft metal on top of them.

**Fig. 11:**
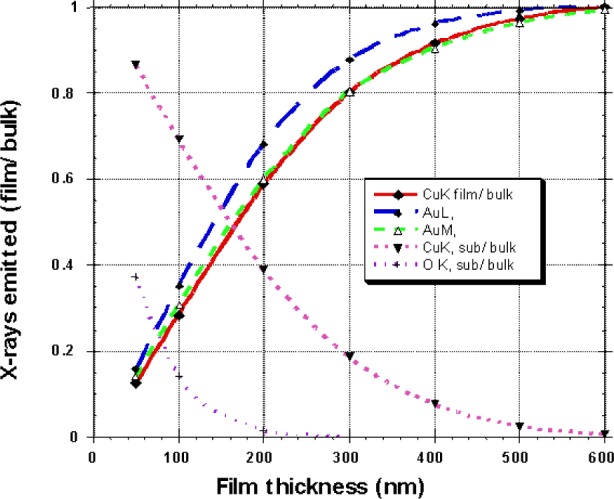
Monte Carlo calculations of emitted x rays for Au20-Cu80 at an electron accelerating potential of 20 keV. For these calculations, we considered a film of composition Au20-Cu80 overtop of a bulk composition of copper oxide. Note that the oxygen signal from the copper oxide substrate is lost when covered by a film thickness of only 200 nm. Also, gold Lα x rays are no longer affected by the copper oxide substrate at a film thickness of 500 nm.

**Fig. 12:**
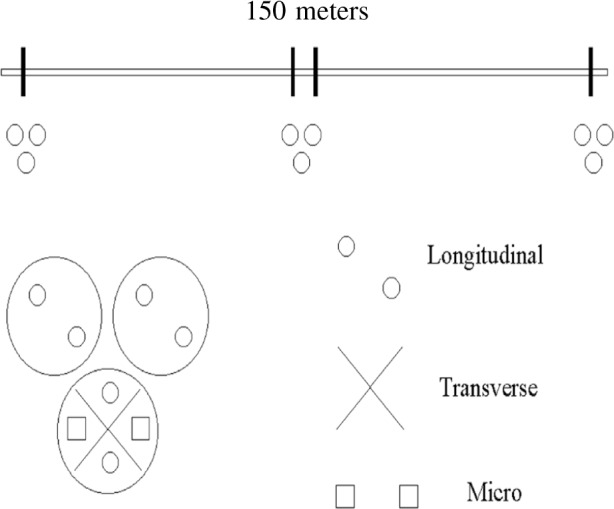
Original homogeneity testing performed before SRM 482 was issued. The wires were sampled at three locations, both ends and one intermediate position. Three cross-sectional metallographic preparations were prepared from each slice. Broad beam (25 μm) longitudinal homogeneity was tested on each cross-section. Transverse and micro-homogeneity were performed on one cross-section from each slice.

When sample preparation techniques result in copper oxide precipitates not being exposed at the surface, they may be difficult to detect using EPMA. Figure 11 represents Monte Carlo calculations for emitted x rays at 20 keV for a film of composition Au20-Cu80 on top of a bulk composition of copper oxide. These calculations indicate that the gold signal from the overlying film is completely unaffected by a copper oxide substrate when the film thickness (Au20-Cu80) is only 500 nm. Similarly, the oxygen signal from the copper oxide substrate is lost when covered by a film thickness of only 200 nm.

## 7. Future Work

Work is in progress to determine copper oxide concentration in wires other than the Au20-Cu80. Bulk chemical analyses will be used to eliminate the difficulty in distinguishing between oxide precipitates and impressed diamond abrasive. Once the concentration of oxide precipitates is known, their effect on the bulk chemical composition of the wires of SRM 482 can be determined. Further EPMA analyses may be performed on other wires of SRM 482 to determine whether oxide precipitates in other compositions are detectable by EPMA.

We are currently working on the development of a sample preparation procedure for SRM 482. Ideally, we prefer to avoid chemical etchants because they may alter the chemistry of these standards. The recommended procedure should retain oxide inclusions while eliminating impressed abrasives.

## 8. Conclusions

Copper oxide precipitates have been observed in all wires of SRM 482 except the pure gold wire. Precipitates are spherically shaped and their size varies with wire composition. They are largest and most easily observed in the Au20-Cu80 wire with sizes ranging from submicrometer up to 2 μm in diameter.

Initial investigations indicate that precipitates might not always be observed in SRM 482. SIMS analyses indicate that inclusions may not be evenly distributed along the length of the wires. Also, the metallographic procedure used to prepare SRM482 for microanalysis plays an important role in determining whether or not inclusions are observed on polished wire surfaces.

Analysis of the Au20-Cu80 wire indicates that the presence of copper oxide precipitates has minimal impact on the use of this wire as a standard for EPMA analysis. Precipitates are small (≤ 2 μm in diameter) and their concentration is low enough (≤ 1 % area) that they are easily avoided during EPMA analyses simply by observing that the beam is hitting the standard in an inclusion free area. When oxide precipitates are avoided, EMPA analyses of the Au20-Cu80 wire agree with NBS certified values.

Even though the impact of copper oxide precipitates on EPMA analyses appears minor, their presence may restrict or preclude the use of some microanalysis techniques. The use of automated analyses routines to check standards should be avoided since this practice may result in the analysis of precipitates rather than the alloy. Oxide precipitates may adversely affect the use of SRM 482 for high spatial resolution microanalysis techniques such as SIMS and Auger electron spectroscopy. Also, avoiding oxide precipitates in the environmental scanning electron microscope (ESEM) will be difficult because of electron beam broadening effects resulting from gaseous molecules in the sample chamber.

## Figures and Tables

**Table 1:** NBS/NIST Certified Values: Au = 20.1_2_ Cu = 79.8_5_^a^. Electron probe microanalysis data for Au20-Cu80 wire

	N^b^		CuKα	CuLα	AuLα	AuMα	O^c^
Spots (precipitates)	11	Average	85.14	85.97	7.01	7.16	6.87
		Std. dev.	1.06	1.26	2.81	2.57	1.42
Wire	36	Average	79.84	80.66	19.56	18.58	
		Std. dev.	0.39	0.33	0.36	0.27	

a All reported values are in mass fraction × 10^2^.

b Number of analyses.

c Oxygen concentration was calculated by difference.

**Table 2:** Preparation procedures

Preparation Procedure #1							

Step	Abrasive	Cloth	Lubricant	Load^a^ N	Time (min)	Rotation direction^b^	Speed^c^ (rpm)
1	400 grit silicon carbide paper		Mineral oil	13	Until planar	Contra	220
2	600 grit silicon carbide paper		Mineral oil	13	1	Contra	220
3	6 μm diamond paste	Nylon	Diamond extender	22	5	Contra	220
4	1 μm diamond paste	Nylon	Diamond extender	22	5	Contra	220
5	0.5 μm diamond suspension	Short nap silk	Odorless Kerosene	13	5	Comp	220

Preparation Procedure #2							

1	400 grit silicon carbide paper		Water	13	Until planar	Contra	220
2	9 μm diamond paste	Texmet 1000	Diamond extender	13	5–10	Comp	220
3	3 μm diamond paste	Texmet 1000	Diamond extender	13	5	Comp	220
4	1 μm diamond paste	Texmet 1000	Diamond extender	13	5	Comp	220
5	0.1 μm diamond paste	Texmet 1000	Diamond extender	13	5	Comp	220

a Load per sample.

b Refers to the direction in which the samples are rotated relative to the rotation direction of the platen surface. Contra means that samples are rotated in a direction contradictory to the rotation direction of the platen. Comp means that the samples are rotated in the same direction as the platen.

c Rotation speed of the platen surface.

## Footnotes

1 NBS was renamed The National Institute of Standards and Technology (NIST) in 1987.

2 Certain commercial equipment, instruments, or materials are identified in this paper to foster understanding. Such identification does not imply recommendation or endorsement by the National Institute of Standards and Technology, nor does it imply that the materials or equipment identified are necessarily the best available for the purpose.

3 Available at www.nist.gov/lispix.

## 9. Appendix A. SRM 482 Certificate of Analysis

### National Bureau of Standards Certificate of Analysis

#### STANDARD REFERENCE MATERIAL 482 Gold-Copper Wires for Microprobe Analysis

These standard reference materials are designed for use in quantitative elemental microprobe analysis. Although the selection of this particular system was circumscribed by the requirements of standard reference materials for electron probe microanalysis, the materials will be equally useful for other micro techniques. Accurate chemical characterization and the achievement of homogeneity on a microscopic scale were given special emphasis.

**Table:**

SRM 482 wire	Color code	Nominal composition	Cominco American^a^	U.S. Bureau of the Mint^b^		NBS^c^		Average value^d^	
			Au	Au	Cu	Au	Cu	Au	Cu
				Percent by weight					
A	Gold	Au 100	–	–	–	–	–	100.0_0_	–
B	Gray	Au80 – Cu20	80.10	80.13	19.81	80.21	19.85	80.1_5_	19.8_3_
C	Yellow	Au60 – Cu40	60.30	60.37	39.66	60.41	39.62	60.3_6_	39.6_4_
D	Blue	Au40 – Cu60	40.12	40.06	59.88	40.11	59.97	40.1_0_	59.9_2_
E	Red	Au20 – Cu80	20.04	20.12	79.84	20.21	79.86	20.1_2_	79.8_5_
F	Copper	Cu100	–	–	–	–	–	–	100.0_0_

a The fire assay method was employed for the determination of Au by Cominco American.

b At the U.S. Bureau of the Mint, Au was determined by fire assay and Cu was determined by electrodeposition.

c At NBS, Au was determined by precipitation from solution; Cu was determined bv electrodeposition.

d The results of individual laboratories agree within a range of ± 0.1% absolute from the average values. The agreement between results by the different methods and analysts, and the summation of results close to 100% for each binary alloy, indicate that the averages are free from significant bias.

The set of standard reference materials, SRM 482, consists of six wires each having a diameter of approximately 0.5 mm and a length of approximately 5 cm. For identification, the four alloy wires were covered with an easily removable colored coating.

The overall direction and coordination of technical measurements leading to certification were performed under the chairmanship of B. F. Scribner.

The technical and support aspects involved in the preparation, certification, and issuance of these standards were coordinated through the Office of Standard Reference Materials by R. E. Michaelis.

**Table:**

Gaithersburg, MD 20899 August 25, 1998 (Revision of certificate dated 6-6-69)	(over)	Stanley D. Rasberry, Chief Office of Standard Reference Materials

##### Preparation and Purity

The standards were prepared by Cominco American, Inc. in the form of wires approximately 150 meters long. The end members of the series, as well as the starting materials for the alloys, were of the highest purity grade and precautions were taken to minimize contamination. Two of the alloy standards were heat-treated at NBS to improve microhomogeneity. The pure metal standards were examined by the residual resistivity ratio technique and the total of electrically active impurities in each was estimated to be about 0,001%. The gold-copper wires were examined spectrographically for metallic impurities; no significant impurities were found at detection limits ranging from 0.0001 to 0.010%.

##### Longitudinal Homogeneity

Variation in composition along the full length of each alloy wire was investigated by electron probe microanalysis for areas 25 *μ*m diameter on cross sections at three positions along the wire including the two ends. The observed differences in composition for the positions, expressed as the range between the highest and lowest values for each alloy, were as follows:

**Table:**

Nominal Composition	Au80	Au60	Au40	Au20
Observed range*	0.3%	0.7%	0.9%	0.9%

Homogeneity along the wires was also tested by measurement of the residual resistivity ratio. These measurements indicated that the variation (macroscopic) of composition along all standard wires was less than 0.1% absolute. Further information on longitudinal homogeneity or the wires was obtained by determinations of Au at the extreme ends of the alloy wires by the Bureau of the Mint; the data also indicate that the extreme variation along the wires is less than 0.1*%* absolute.

##### Transverse and Micro Homogeneity

Variation in composition within the above mentioned cross sections of the wires was investigated by electron probe microanalysis. For each cross section, measurements were made along two diagonals at right angles. On each diagonal, determinations were made at 25 points, 1 *μ*m or less in diameter, starting and ending at approximately 25 *μ*m from the edge. For each alloy, the element which could be determined with the better precision was used in the evaluation. The variation was calculated in terms of the standard deviation for an individual determination for each traverse. In the table below, the variation is presented as the range between the lowest and highest observed standard deviations for the six traverses performed on each alloy.

**Table:**

Nominal Composition	Element Determined	Range of Standard Deviations for Traverses*
Au80	Cu	0.09 – 0.24%
Au60	Cu	.16 – .27
Au40	Au	.13 – .23
Au20	Au	.13 – .20

The homogeneity on a microscopic scale was further investigated by performing quantitative measurements in two arrays of 10 × 10 points (1 *μ*m diameter) on each of the cross sections. The distance between adjacent points was 3.5 μm. This was repeated on several cross sections so that 6 arrays were obtained on each alloy. For the element which could be measured with better precision, the range is given between the lowest and highest observed standard deviation for an individual determination for the 6 arrays for each alloy.

**Table:**

Nominal Composition	Element Determined	Range of Standard Deviations for Arrays*
Au80	Cu	0.19 – 0.28%
Au60	Cu	.28 – .37
Au40	Au	.25 – .31
Au20	Au	.12 – .20

* The ranges indicated are close to the precision of the method and should he considered upper limits of estimates of inhomogeneity.

Extensive homogeneity studies were performed with the electron probe microanalyzer at NBS by M. A. Giles, D. L. Vieth, R. L. Myklebust, C. E. Fiori, and K. F. J. Heinrich. Measurements of residual resistivity ratio were made at NBS, Boulder, Colorado, by R. L. Rutter and R. L. Powell. Heat treatment of the alloys at NBS was performed by G. E. Hicho and M. R. Meyerson. Spectrographic survey analyses were made at NBS by V. C. Stewart. Determinations of composition were made at Cominco American, Inc., Spokane, Washington, by T. A. Rice; at the U. S. Bureau of the Mint, Washington, D. C., by H. G. Hanson, Jr.; and at NBS by J. R. Baldwin and R. A. Durst.

## 10. Appendix B. Summary of Original Homogeneity Testing

The original homogeneity testing is thoroughly discussed by Heinrich and co-authors [1]. Original homogeneity testing is summarized in this appendix because it pertains to the current study and also because the original reference (published over 30 years ago and now out of print) may be difficult to obtain.

For SRM 482, EPMA homogeneity testing was performed at 3 locations along the length of each wire. These three locations included both ends and one intermediate position. At each of these three locations, three cross-section samples were metallographically prepared (Fig. 12). Homogeneity analyses consisted of three different measurements referred to as longitudinal homogeneity, transverse homogeneity and micro-homogeneity. Longitudinal homogeneity was measured on all metallographically prepared cross-sections while transverse and micro homogeneity was measured on one cross-section at each of the three locations sampled (Fig. 12). Chemical composition of the wire was determined for longitudinal homogeneity whereas for transverse and micro homogeneity, x-ray count data was used for homogeneity determinations. Longitudinal homogeneity consisted of a broad beam (25 μm diameter) electron probe analysis conducted in opposite quadrants of the cross-section. Transverse homogeneity consisted of data points collected along two diagonals at right angles. Beam size was one micrometer or less in diameter. Twenty-five data points were collected along each diagonal starting and ending approximately 25 μm from the edge of the wire (Fig. 12). For micro-homogeneity, quantitative data was collected from a two dimensional array of 10 × 10 points. The beam diameter was 1 μm and the distance between adjacent points was 3.5 μm. Micro-homogeneity was also tested in opposite quadrants of the cross-section. A total of over 750 EPMA data points were collected to test the homogeneity of each wire in SRM 482 [1]. The results of this homogeneity testing are listed in the certificate of analysis (Appendix A).

## 11. Appendix C. Mechanical Grinding and Polishing Procedures

Work is in progress to develop a recommended procedure for the sample preparation of SRM 482. Since SRM 482 is a standard for chemical microanalysis, the use of chemical etchants during sample preparation is not recommended. Etchants may alter the certified chemical composition of this standard.

Two different preparation procedures are listed in Table 2 of this Appendix to clarify text and figures within this manuscript. Neither procedure is ideal or recommended by the authors. Procedure #1 is good for the retention/observation of oxide inclusions but the finished surface is scratched and diamond abrasives are impressed. In procedure #2, inclusions are lost and the abrasive is heavily impressed into these samples.

We have observed that the use of fine alumina suspensions (0.05 μm) on napped cloths (dense short napped nylon or silk) under a fairly high load (30 N) improves the surface finish of theses wires by reducing scratches and impressed abrasives. Initial experience indicates, however, that the use of alumina may degrade the visibility of the copper oxide inclusions. Suspensions of colloidal silica, when applied as a final polishing step, degraded the surface polish of these wires.
